# Supplementary material for: eQTL of KCNK2 regionally influences the brain sulcal widening: evidence from 15,597 UK Biobank participants with neuroimaging data
Source: Brain Struct Funct. 2018 Dec 5;224(2):847–57. doi: 10.1007/s00429-018-1808-9 (PMC6420450; doi:10.1007/s00429-018-1808-9)
Supplement: Supplementary file 1 — Supplementary material 1 (PDF 4842 KB) [file 429_2018_1808_MOESM1_ESM.pdf]

Table S1. Associated slopes for the Figure 1. (c)

Feature	Sulcus	Hemisphere	Slope
Opening	Sylvian Fissure	left	0.0322
		right	0.0325
	Anterior Cingulate	left	0.0217
		right	0.0228
	Posterior Cingulate	left	0.0282
		right	0.0277
	Intraparietal	left	0.0252
		right	0.0232
	Central	left	0.029
		right	0.0293
	Inferior Frontal	left	0.0244
		right	0.0225
	Superior Frontal	left	0.0254
		right	0.0252
	Inferior Temporal	left	0.0145
		right	0.014
	Superior Temporal	left	0.0259
		right	0.0261
	Subparietal	left	0.02
		right	0.0206
Grey Matter Thickness	Sylvian Fissure	left	-0.019
		right	-0.0183
	Anterior Cingulate	left	-0.0181
		right	-0.0199
	Posterior Cingulate	left	-0.0125
		right	-0.0139
	Intraparietal	left	-0.0095
		right	-0.012
	Central	left	-0.0111
		right	-0.0105
	Inferior Frontal	left	-0.0145
		right	-0.0126
	Superior Frontal	left	-0.0105
		right	-0.0099
	Inferior Temporal	left	-0.0154
		right	-0.0156
	Superior Temporal	left	-0.0199
		right	-0.0194
	Subparietal	left	-0.0207
		right	-0.0244

Table S2. Heritability estimates and their associated p-values corresponding to Figure 2.

Feature	Sulcus	Left Hemisphere	Right Hemisphere
Opening	Sylvian Fissure	0.4 ± 0.048 ($5.3 \cdot 10^{-18}$)	0.423 ± 0.049 ($5.3 \cdot 10^{-19}$)
	Anterior Cingulate	0.23 ± 0.049 ($1.1 \cdot 10^{-6}$)	0.099 ± 0.048 (0.02)
	Posterior Cingulate	0.275 ± 0.049 ($7.2 \cdot 10^{-9}$)	0.19 ± 0.049 ($4.9 \cdot 10^{-5}$)
	Intraparietal	0.388 ± 0.049 ($1.6 \cdot 10^{-15}$)	0.345 ± 0.049 ($4.8 \cdot 10^{-13}$)
	Central	0.445 ± 0.049 ($1.7 \cdot 10^{-19}$)	0.349 ± 0.049 ($6.7 \cdot 10^{-13}$)
	Inferior Frontal	0.269 ± 0.049 ($1.2 \cdot 10^{-8}$)	0.317 ± 0.048 ($9.7 \cdot 10^{-12}$)
	Superior Frontal	0.246 ± 0.049 ($3.2 \cdot 10^{-7}$)	0.285 ± 0.049 ($2.4 \cdot 10^{-9}$)
	Inferior Temporal	0.151 ± 0.049 ($9.2 \cdot 10^{-4}$)	0.184 ± 0.049 ($6.6 \cdot 10^{-5}$)
	Superior Temporal	0.342 ± 0.048 ($2.4 \cdot 10^{-13}$)	0.318 ± 0.049 ($1.2 \cdot 10^{-11}$)
	Subparietal	0.198 ± 0.049 ($2.8 \cdot 10^{-5}$)	0.175 ± 0.048 ($1.1 \cdot 10^{-4}$)
Grey Matter Thickness	Sylvian Fissure	0.371 ± 0.048 ($2.8 \cdot 10^{-16}$)	0.323 ± 0.048 ($1.5 \cdot 10^{-12}$)
	Anterior Cingulate	0.218 ± 0.049 ($3.1 \cdot 10^{-6}$)	0.079 ± 0.048 (0.05)
	Posterior Cingulate	0.144 ± 0.049 ($1.3 \cdot 10^{-3}$)	0.127 ± 0.048 ($3.4 \cdot 10^{-3}$)
	Intraparietal	0.173 ± 0.048 ($1.0 \cdot 10^{-4}$)	0.239 ± 0.048 ($2.0 \cdot 10^{-7}$)
	Central	0.232 ± 0.048 ($4.2 \cdot 10^{-7}$)	0.253 ± 0.049 ($5.6 \cdot 10^{-8}$)
	Inferior Frontal	0.223 ± 0.048 ($1.2 \cdot 10^{-6}$)	0.224 ± 0.049 ($1.6 \cdot 10^{-6}$)
	Superior Frontal	0.151 ± 0.048 ($6.3 \cdot 10^{-4}$)	0.167 ± 0.048 ($2.0 \cdot 10^{-4}$)
	Inferior Temporal	0.1 ± 0.048 (0.02)	0.166 ± 0.049 ($2.6 \cdot 10^{-4}$)
	Superior Temporal	0.254 ± 0.048 ($2.9 \cdot 10^{-8}$)	0.266 ± 0.048 ($7.4 \cdot 10^{-9}$)
	Subparietal	0.159 ± 0.049 ($5.7 \cdot 10^{-4}$)	0.123 ± 0.048 ($4.9 \cdot 10^{-3}$)

Table S3. Summary GTEx information on the significant loci (GTEx Analysis Release V7 (dbGaP Accession phs000424.v7.p2))

**chr**	**rsid**	**maf in ~12k subjects**	**Nearest gene(s)**	**eQTLs GTEx (meta p-val)**
1	rs864736	0.45765	*KCNK2*	*KCNK2* (multi-tissue 9.7*10^-6; significant tissue: ovary)
1	rs59084003	0.06957	*KCNK2*	No significant eQTLs (maf probably too low)
8	rs11774568	0.27672	*DEFB136; DEFB135*	Highly intergenic regions, notably significant eQTLs in brain tissues with genes [*'CTSB', 'RP11-481A20.10', 'RP11-481A20.11', 'RP11-351I21.7', 'RP11-351I21.6', 'FAM66A'*]
9	rs10980645	0.29548	Intron of *LPAR1*	No significant eQTLs
12	rs12146713	0.09496	Intron of *NUAK1*	ENSG00000257890.1 (lincRNA) (multi-tissue 4.1*10^-28; significant tissues: skin-lower_leg; artery-tibial; adipose-subcutaneous)
16	rs9933149	0.37965	*LOC101928708; C16orf95; FBXO31*	ENSG00000261651.1 (antisense RNA) (p-val computed only in testis tissue 3.0*10^-7)

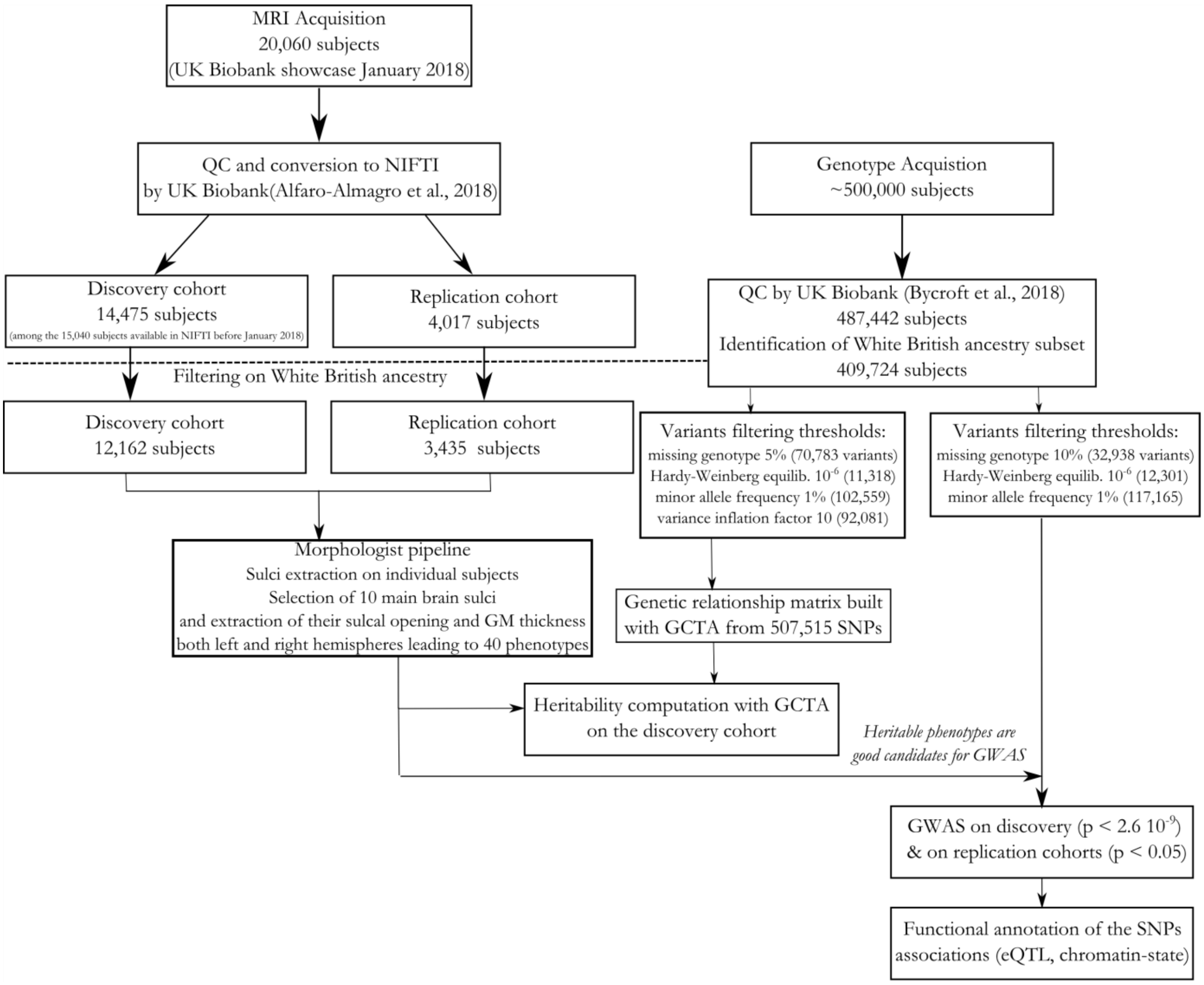

Figure S1. Schematic of the study design.

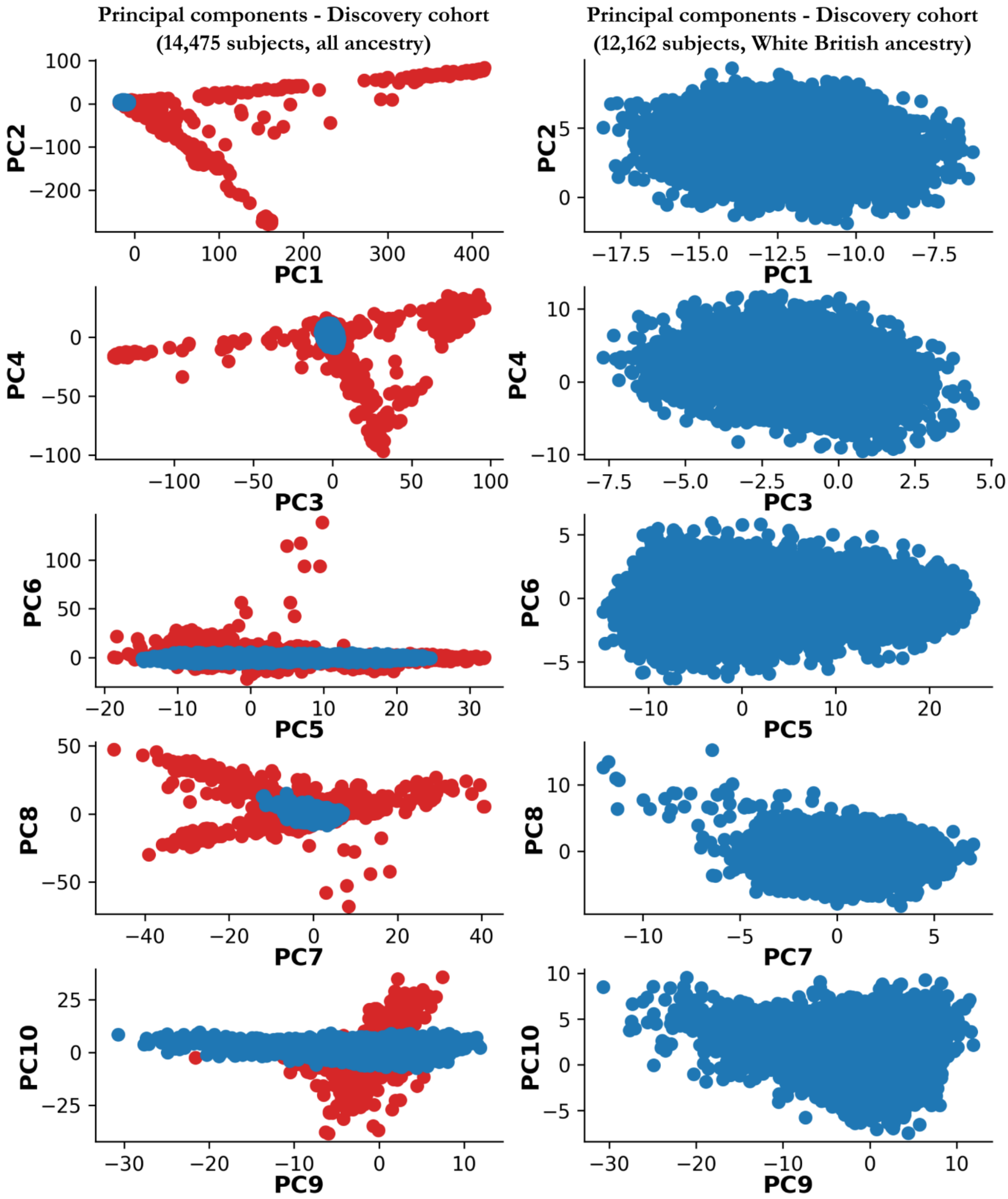

Figure S2. Ten genetic principal components (PCs) provided by UK Biobank to account for population stratification.

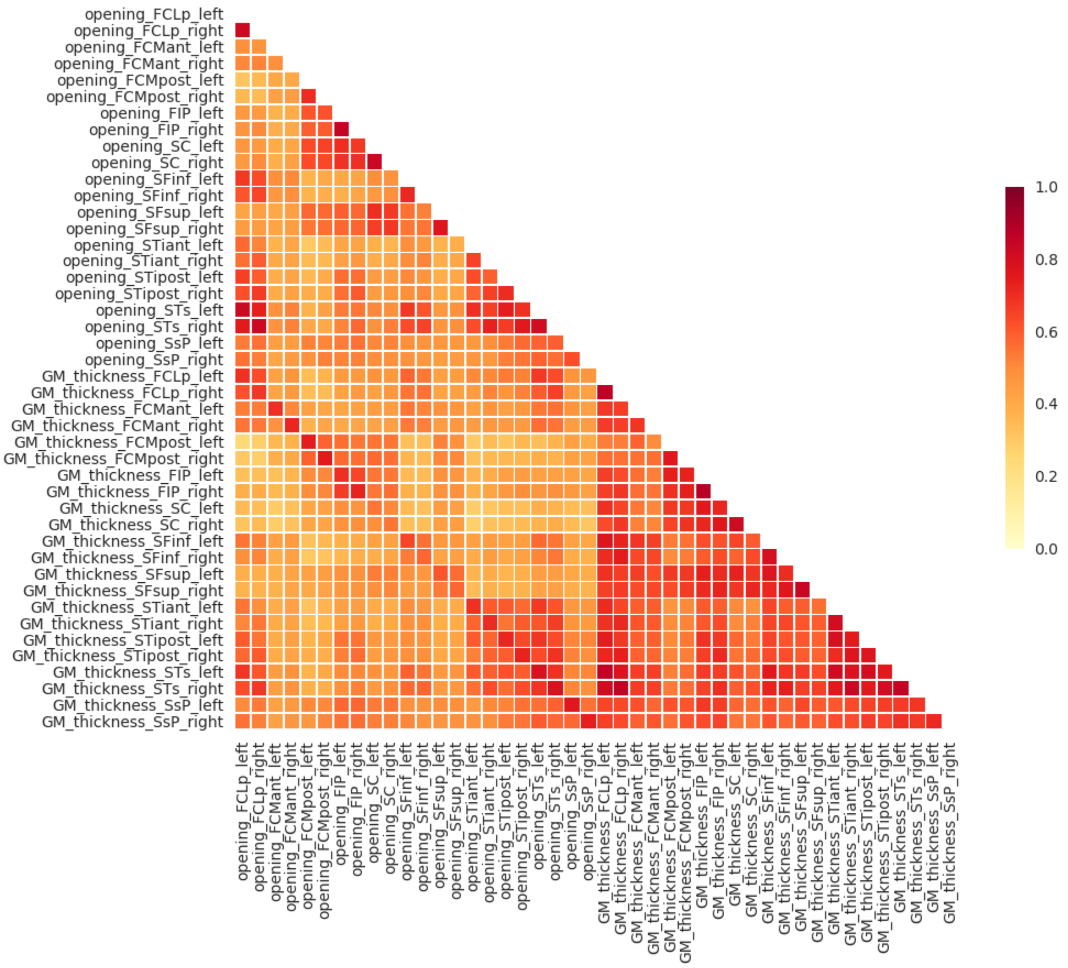

Figure S3. Correlation matrix (absolute values) of the 40 phenotypes considered. Note that, without taking the absolute value, opening vs GM thickness correlations are always negative, while opening and GM thickness separately are always positively correlated.

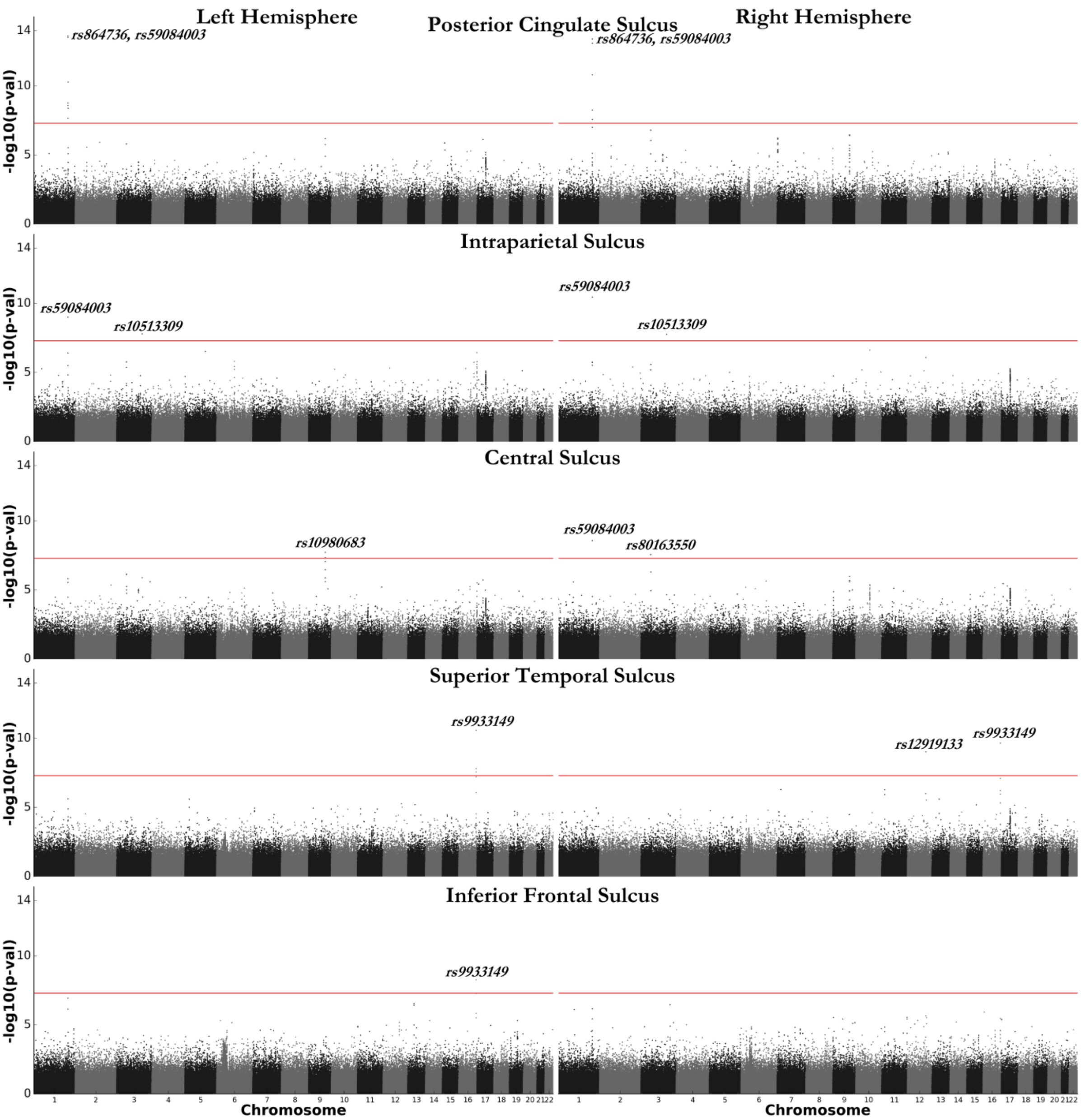

Figure S4. Manhattan plots for five sulci considering their opening as the phenotype for the GWAS.

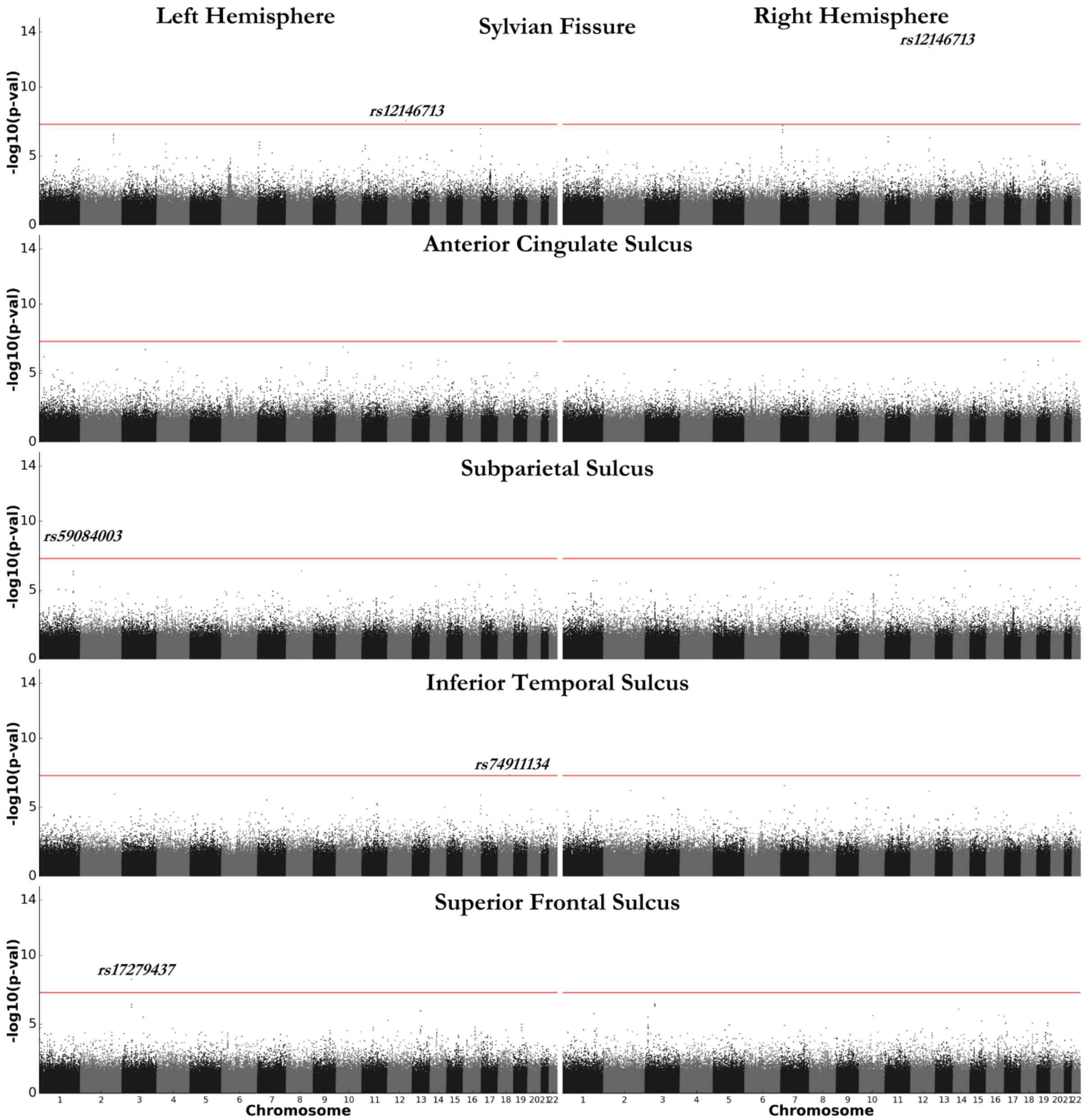

Figure S5. Manhattan plots for five sulci considering their opening as the phenotype for the GWAS.

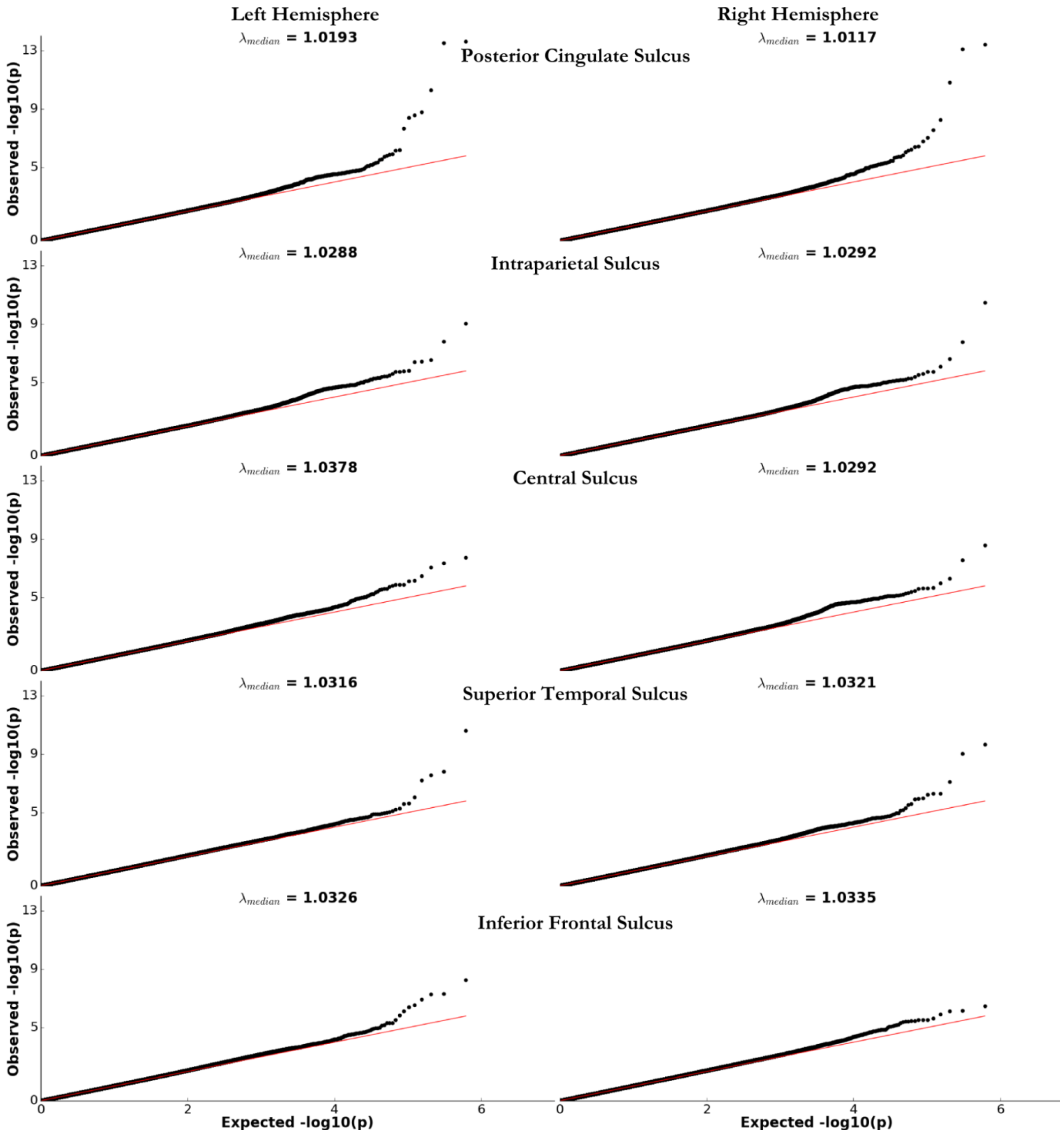

Figure S6. QQ plots for five sulci considering their opening as the phenotype for the GWAS.

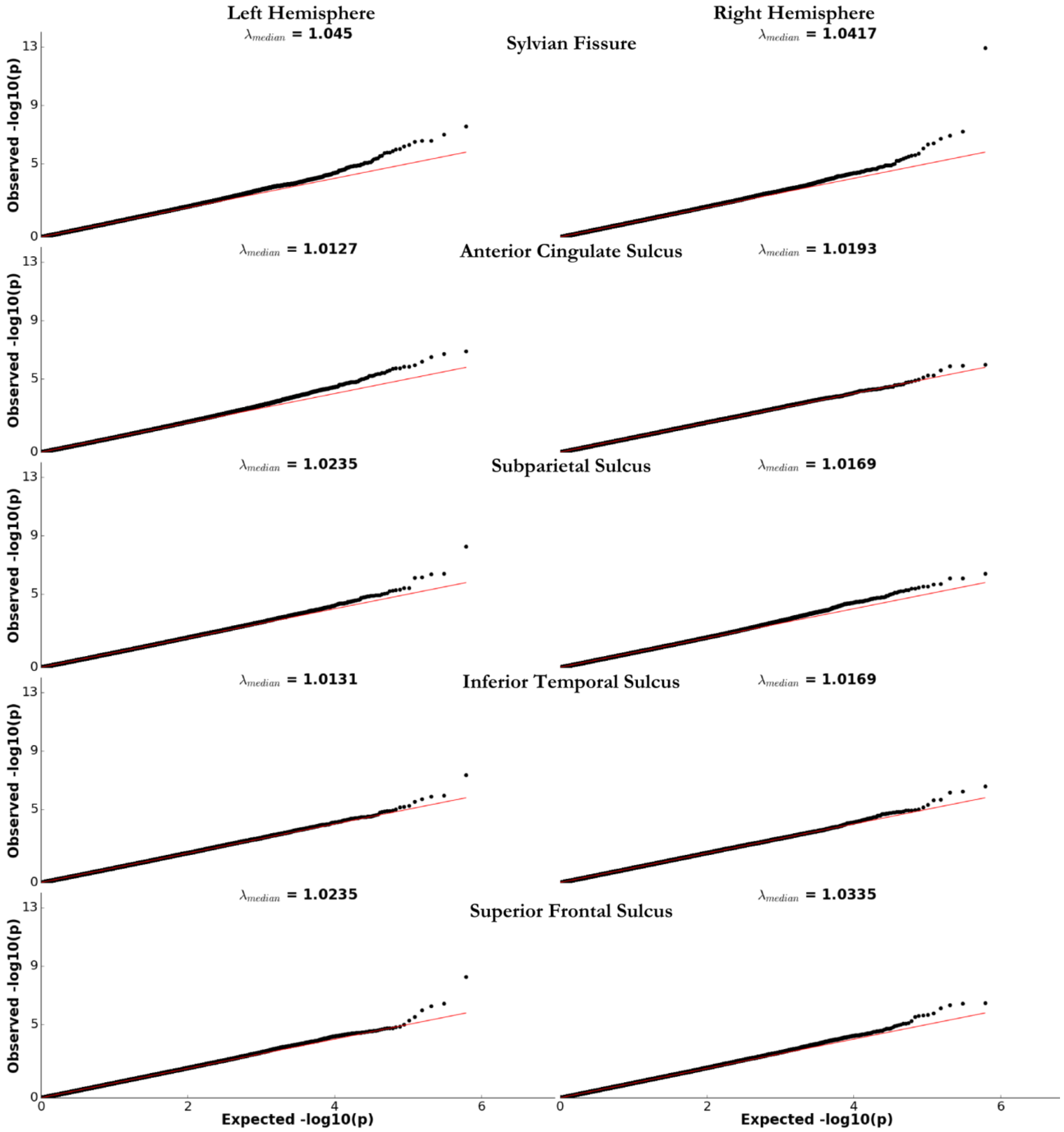

Figure S7. QQ plots for five sulci considering their opening as the phenotype for the GWAS.

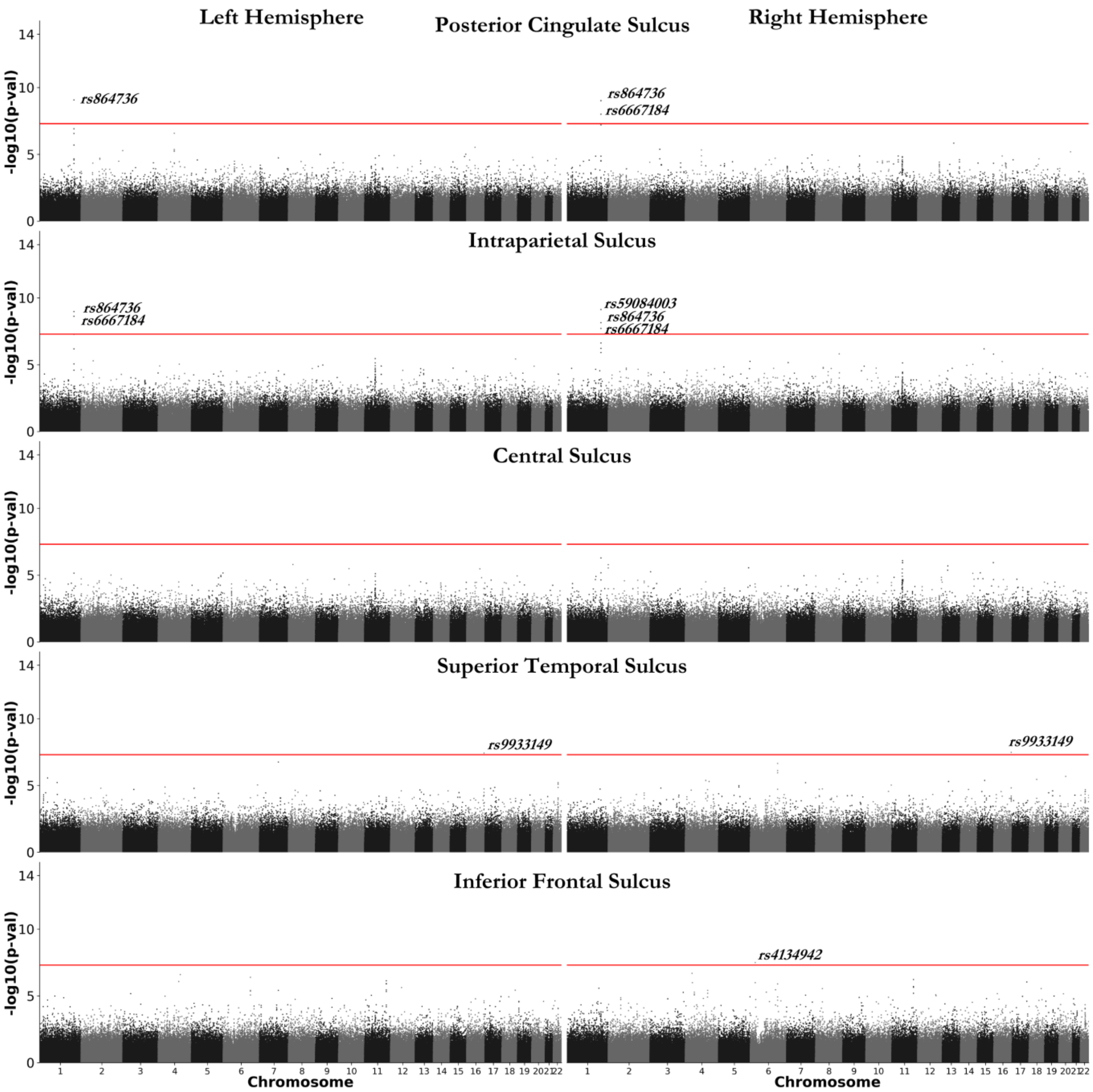

Figure S8. Manhattan plots for five sulci considering their GM thickness as the phenotype for the GWAS.

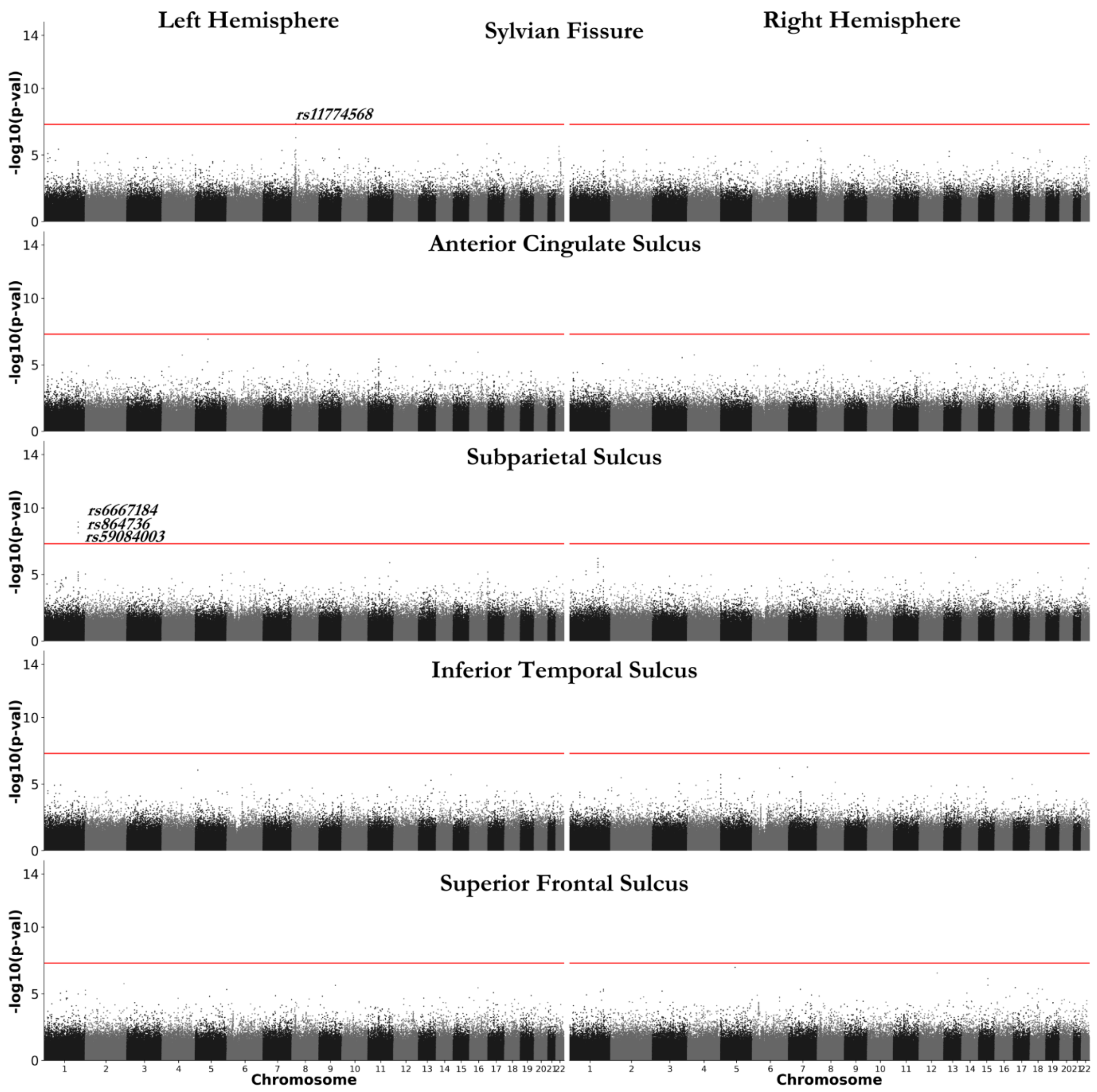

Figure S9. Manhattan plots for five sulci considering their GM thickness as the phenotype for the GWAS.

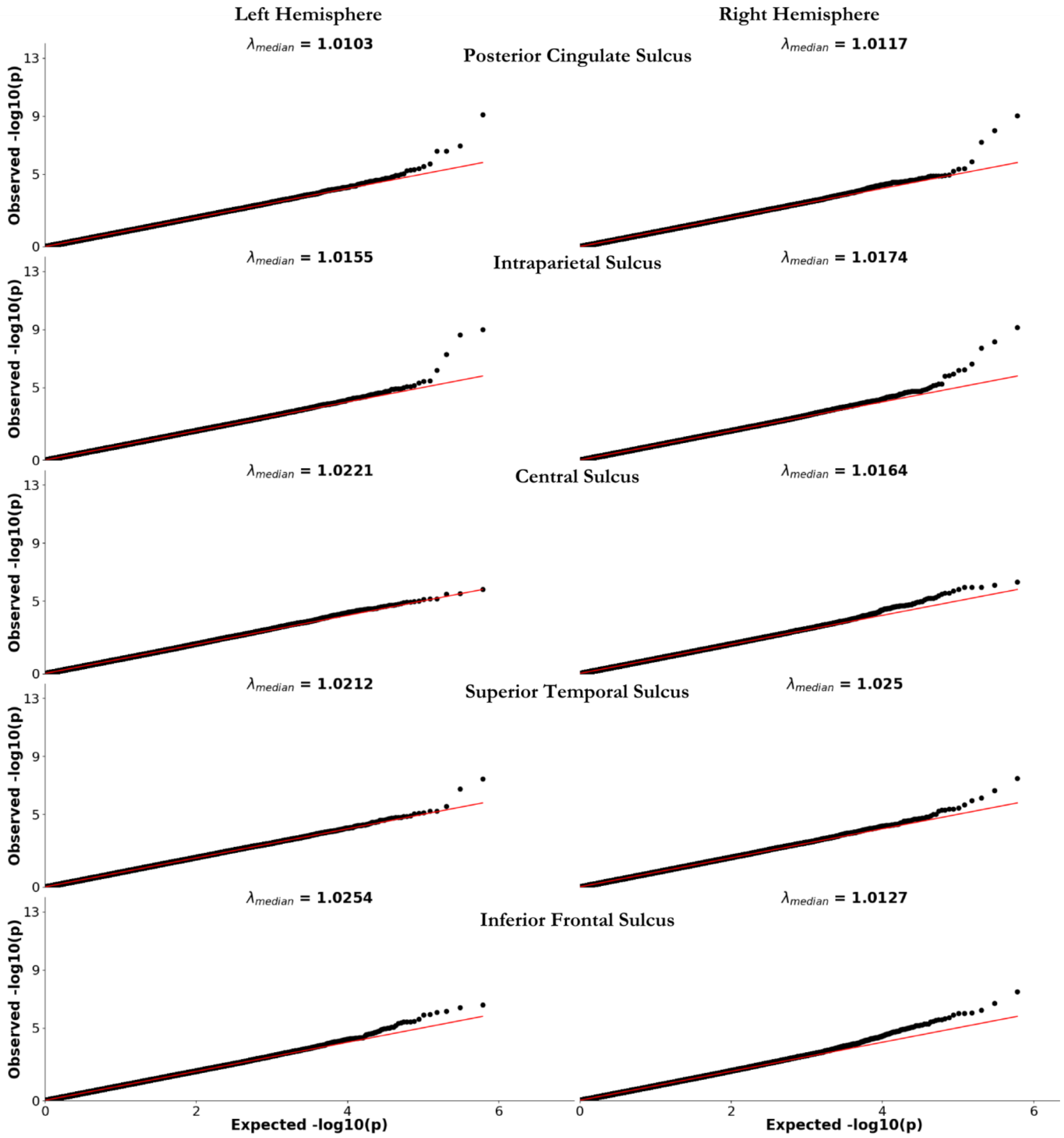

Figure S10. QQ plots for five sulci considering their GM thickness as the phenotype for the GWAS.

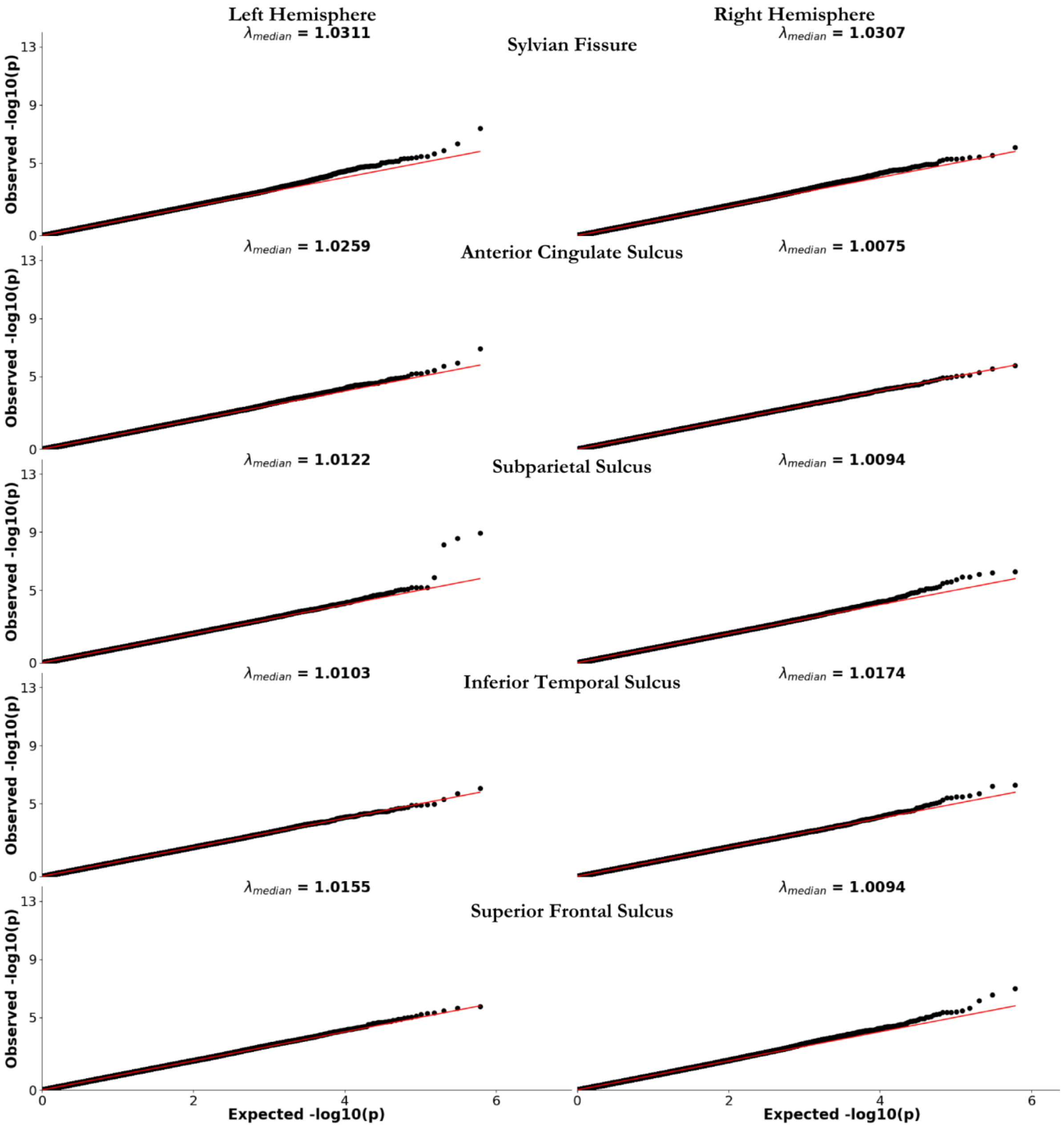

Figure S11. QQ plots for five sulci considering their GM thickness as the phenotype for the GWAS.

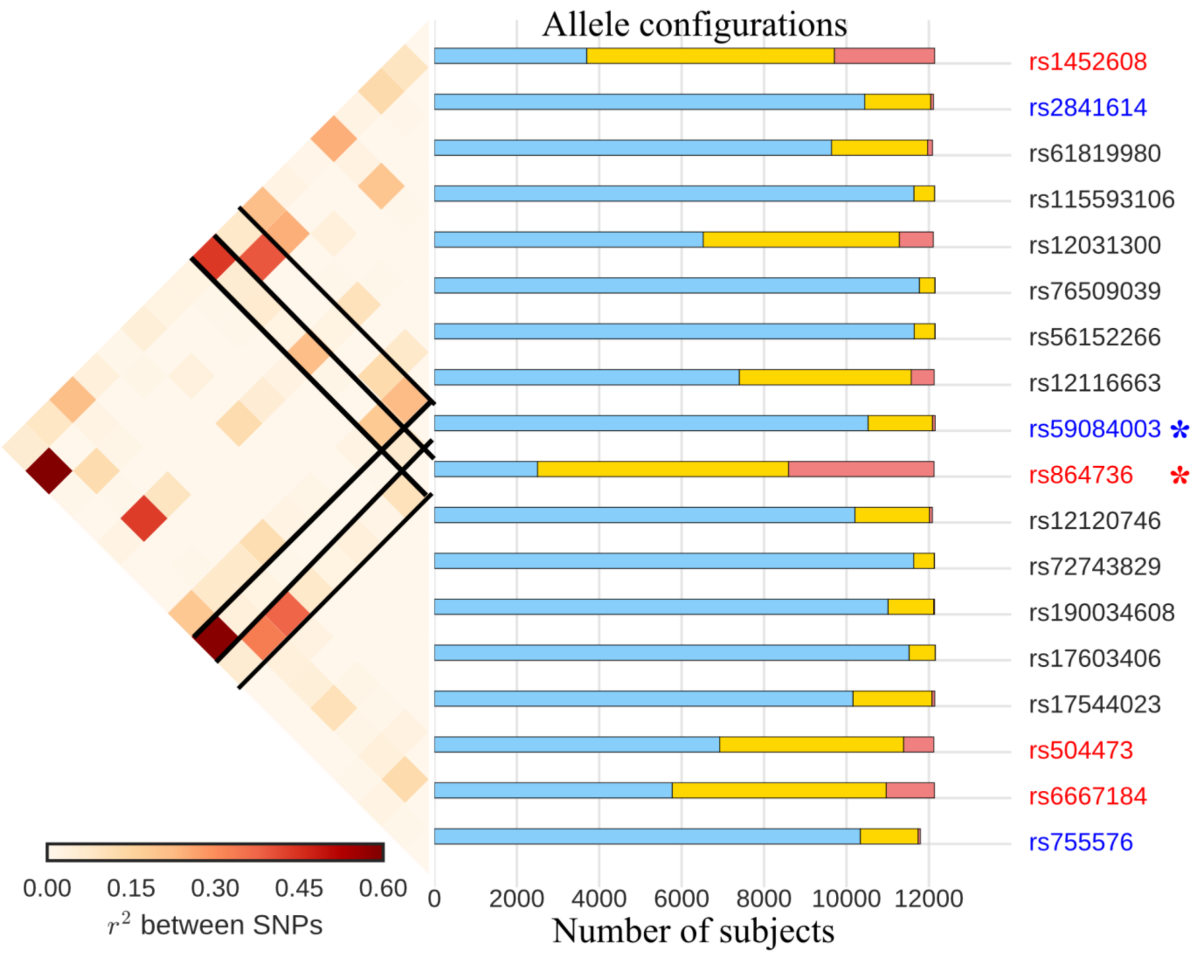

Figure S12. Linkage disequilibrium (r^2 computed with PLINK) for SNPs in the significant locus upward of *KCNK2*. The number of subjects for the different allelic configurations is displayed (number of subjects homozygote major allele in blue, heterozygote in yellow, homozygote minor allele in red). The significant variants in GWAS (**Tab. 1**) in LD with rs864736 and rs59084003 are in red and blue, respectively.

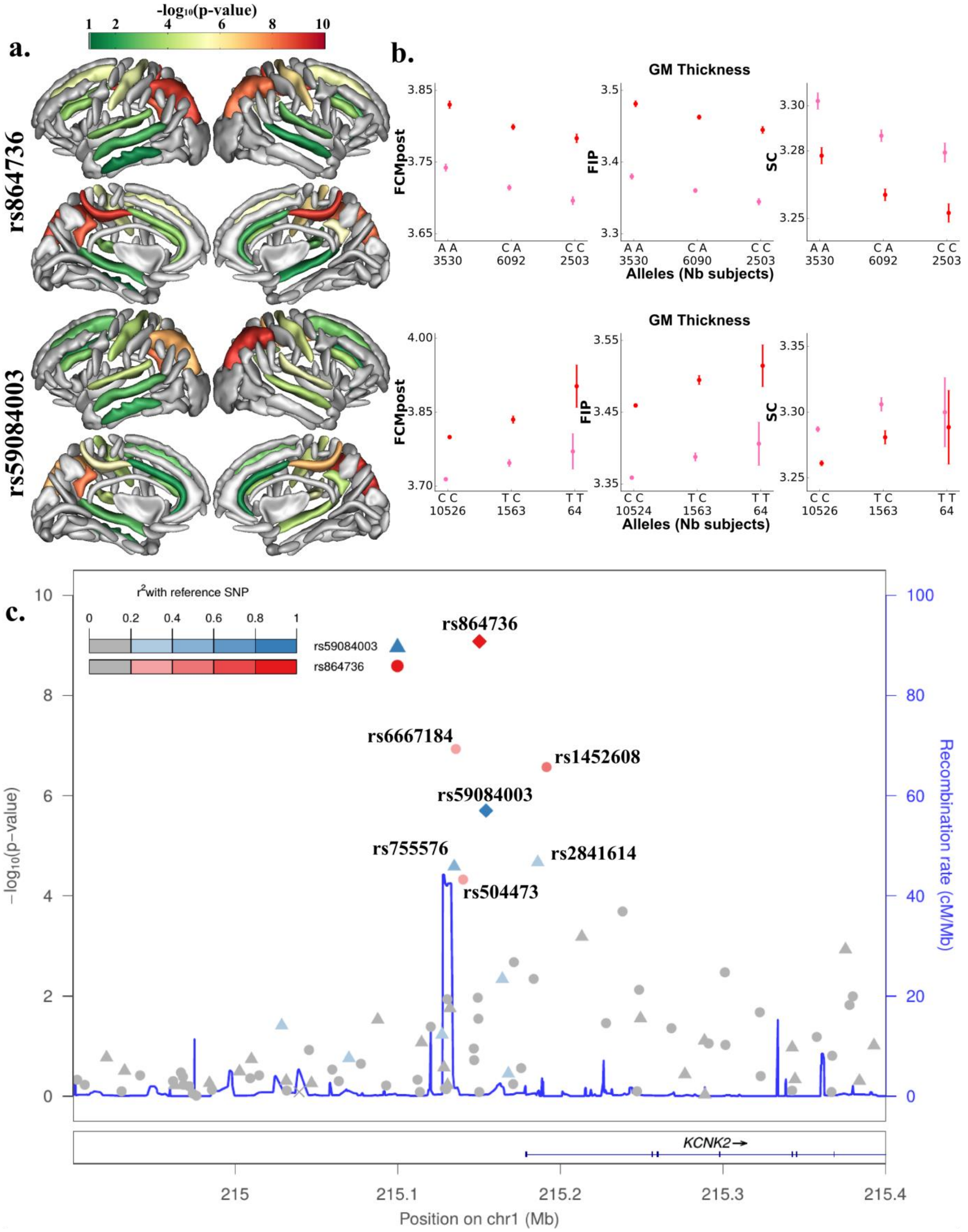

Figure S13. Most significant GWAS hits on *KCNK2* regulating the GM thickness. First and second lines correspond to rs864736 and rs59084003, respectively. Lines represents respectively: **a.** the log10(p-value) of each SNPs mapped onto the nominally significant sulci among the ten considered; **b.** the mean GM thickness and standard error for each configuration of variants in the most significant sulci; **c.** Locuszoom display (Pruim et al. 2011) of the phenotype-variants association for the region upward to *KCNK2* with the left posterior cingulate sulcus opening as a phenotype..

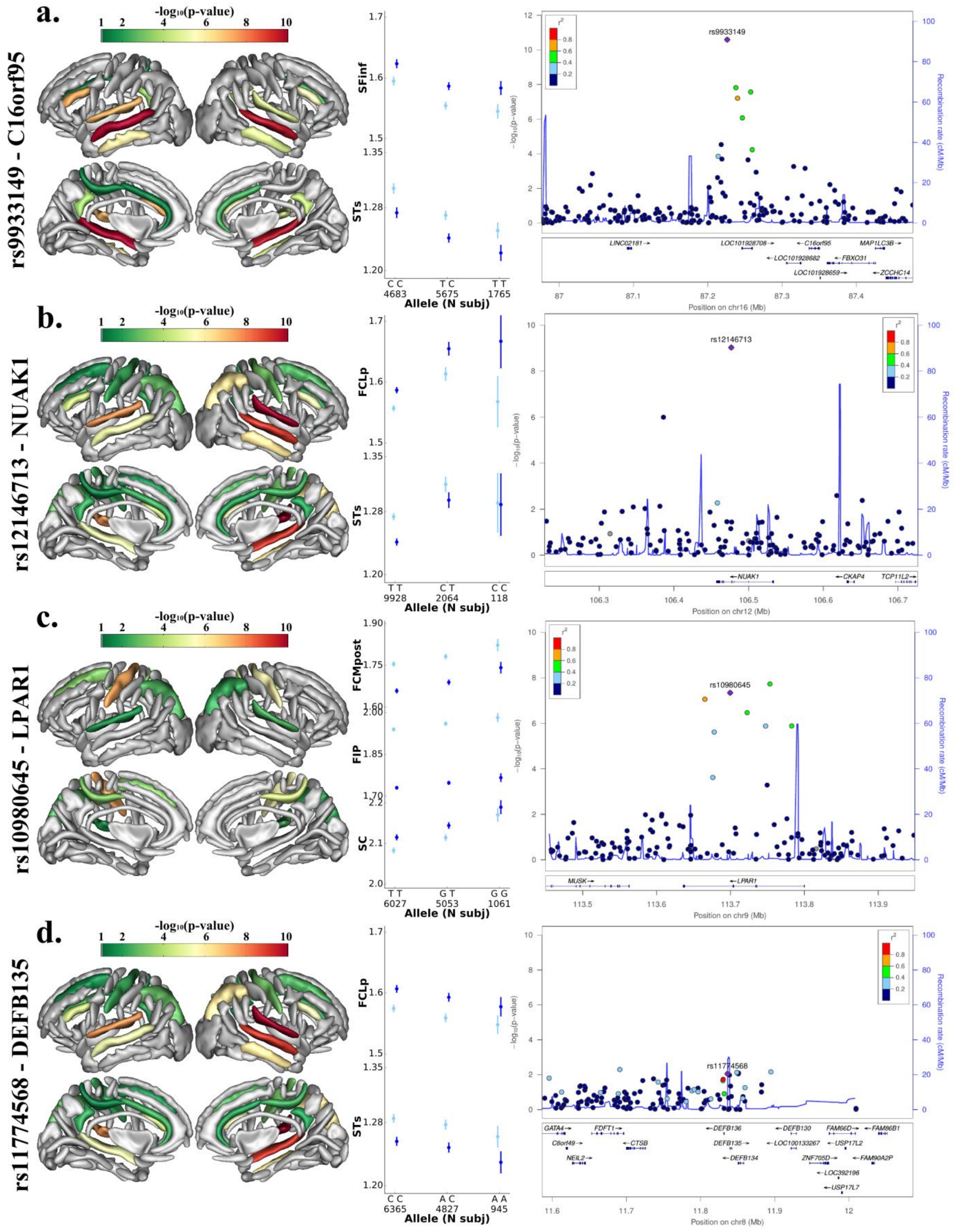

Figure S14. Four significant GWAS hits on the sulcal opening. Lines correspond to the SNPs presented in the following order rs9933149 (**a.**), rs12146713 (**b.**) rs10980645 (**c.**) rs11774568 (**d.**). Columns represents respectively the log10(p-value) of each SNPs mapped onto the nominally significant sulci among the ten considered; the mean sulcal opening and standard error for each configuration of variants in the most significant sulci; Locuszoom display (Pruim et al. 2011) for each variant with the associated most significant phenotype.

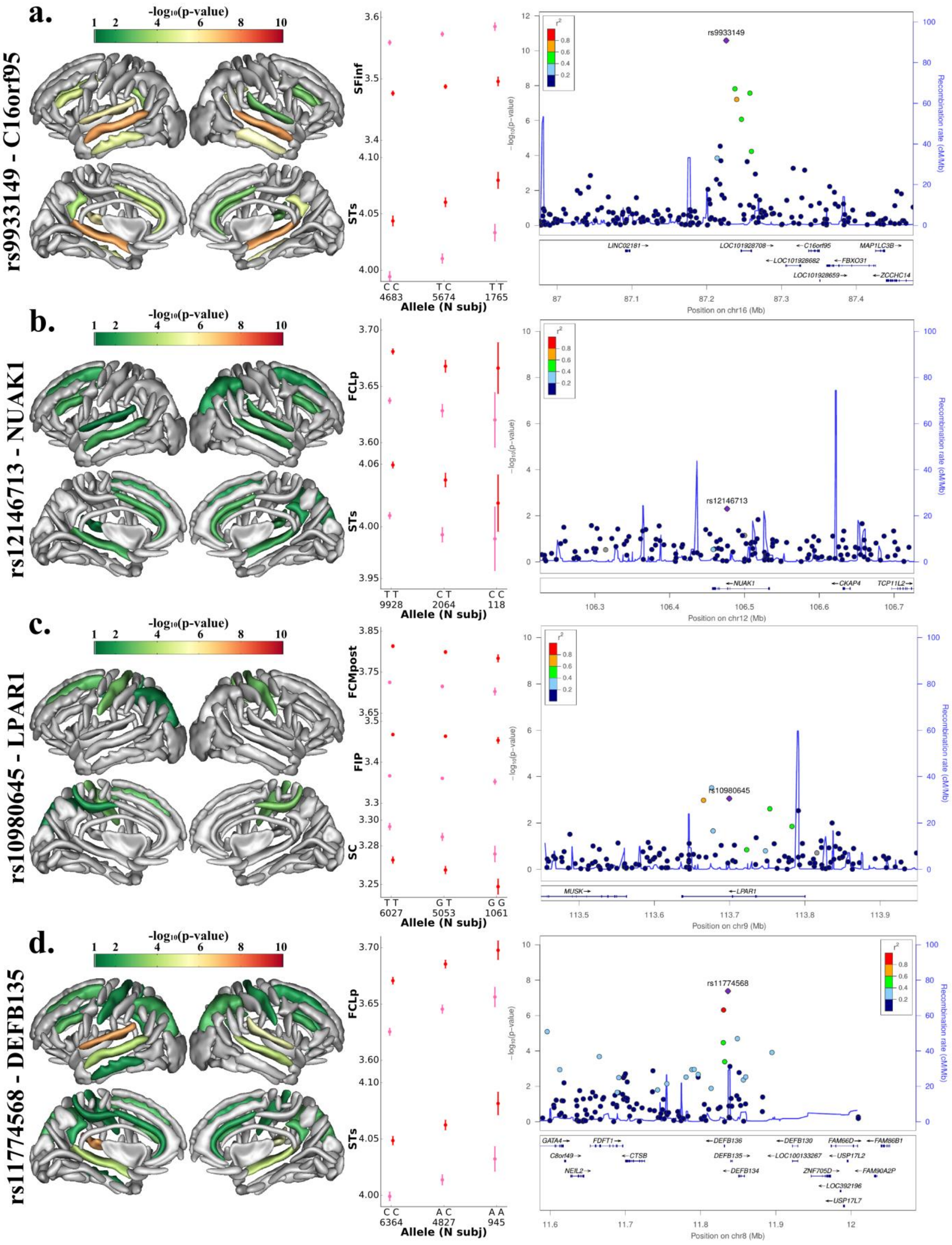

Figure S15. Four significant GWAS hits on the grey matter thickness of the sulci. Lines correspond to the SNPs presented in the following order rs9933149 (**a.**), rs12146713 (**b.**) rs10980645 (**c.**) rs11774568 (**d.**). Columns represents respectively: the log10(p-value) of each SNPs mapped onto the nominally significant sulci among the ten considered; the mean sulci grey matter thickness and standard error for each configuration of variants in the most significant sulci; Locuszoom display (Pruim et al. 2011) for each variant with the associated most significant phenotype.

Tissue	Samples	Beta	p-value
Brain - Substantia nigra	80	0.243	0.04
Brain - Cerebellar Hemisphere	125	0.178	0.1
Brain - Cortex	136	0.162	0.1
Brain - Hypothalamus	108	0.160	0.08
Adipose - Subcutaneous	385	0.155	1.6e-3
Nerve - Tibial	361	0.151	2.5e-3
Uterus	101	0.139	0.2
Colon - Transverse	246	0.130	0.01
Vagina	106	0.130	0.4
Brain - Anterior cingulate cortex (BA24)	109	0.120	0.2
Heart - Left Ventricle	272	0.106	0.2
Breast - Mammary Tissue	251	0.104	0.04
Brain - Hippocampus	111	0.0873	0.3
Testis	225	0.0850	0.3
Adrenal Gland	175	0.0714	0.08
Stomach	237	0.0696	0.3
Esophagus - Mucosa	358	0.0615	0.3
Brain - Amygdala	88	0.0488	0.6
Thyroid	399	0.0480	0.3
Brain - Nucleus accumbens (basal ganglia)	130	0.0466	0.5
Muscle - Skeletal	491	0.0460	0.3
Pancreas	220	0.0451	0.6
Artery - Tibial	388	0.0450	0.4
Skin - Sun Exposed (Lower leg)	414	0.0426	0.4
Artery - Aorta	267	0.0399	0.6
Lung	383	0.0274	0.6
Esophagus - Gastroesophageal Junction	213	0.0222	0.8
Small Intestine - Terminal Ileum	122	0.0171	0.8
Colon - Sigmoid	203	0.0142	0.9
Brain - Caudate (basal ganglia)	144	0.0135	0.7
Cells - Transformed fibroblasts	300	0.00545	0.9
Minor Salivary Gland	85	0.00131	1
Prostate	132	-0.0162	0.9
Brain - Frontal Cortex (BA9)	118	-0.0165	0.8
Brain - Cerebellum	154	-0.0198	0.8
Esophagus - Muscularis	335	-0.0217	0.7
Brain - Putamen (basal ganglia)	111	-0.0234	0.6
Artery - Coronary	152	-0.0256	0.7
Skin - Not Sun Exposed (Suprapubic)	335	-0.0467	0.4
Adipose - Visceral (Omentum)	313	-0.0549	0.4
Pituitary	157	-0.0702	0.4
Brain - Spinal cord (cervical c-1)	83	-0.100	0.3
Ovary	122	-0.501	6.5e-6

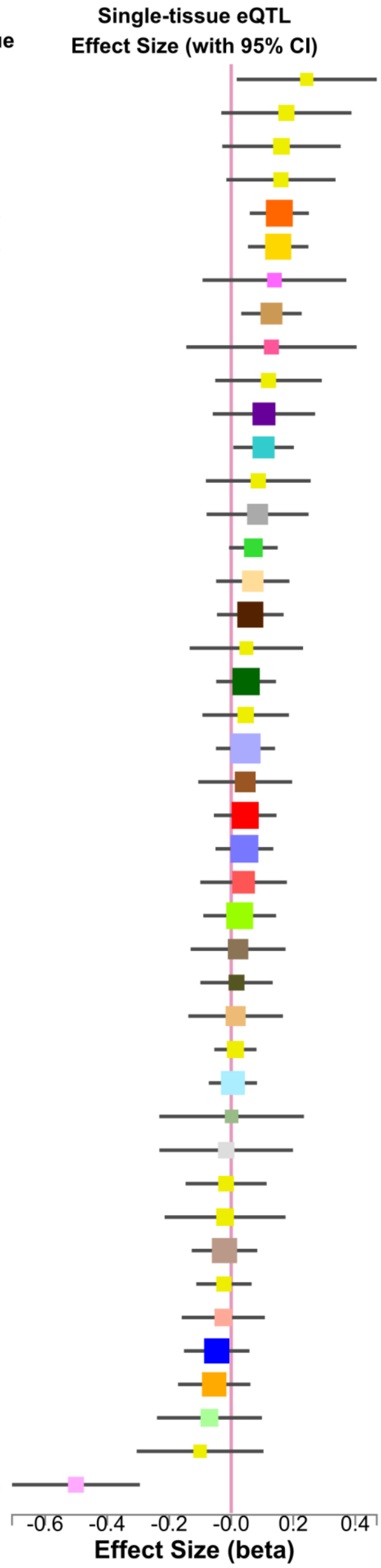

Figure S16. Multi-tissue eQTLs comparison for gene *KCNK2* and variant rs864736 (ENSG00000082482.9 *KCNK2* and 1_215150260_C_A_b37 eQTL). Meta-Analysis Random Effect Model2 (Han and Eskin 2011) p-val = $9.7 \cdot 10^{-6}$. (Data Source: GTEx Analysis Release V7, dbGaP Accession phs000424.v7.p2)
